# Janus Droplet Formation via Thermally Induced Phase Separation: A Numerical Model with Diffusion and Convection

**DOI:** 10.1021/acs.langmuir.2c00308

**Published:** 2022-05-26

**Authors:** Haodong Zhang, Fei Wang, Britta Nestler

**Affiliations:** † Institute of Applied Materials-Microstructure Modelling and Simulation, Karlsruhe Institute of Technology (KIT), Straße am Forum 7, 76131 Karlsruhe, Germany; ‡ Institute of Digital Materials Science, Karlsruhe University of Applied Sciences, Moltkestraße 30, 76133 Karlsruhe, Germany

## Abstract

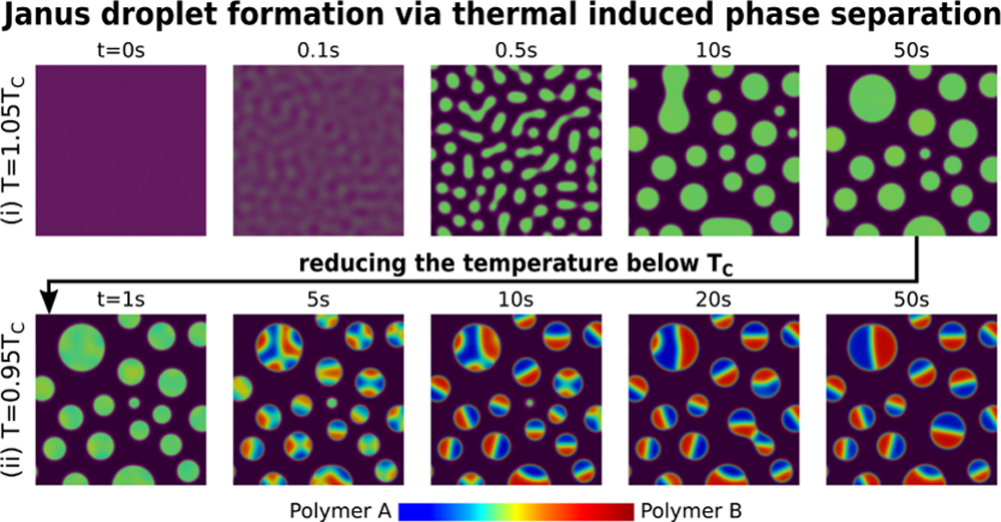

Microscale Janus particles have versatile potential applications in many physical and biomedical fields, such as microsensor, micromotor, and drug delivery. Here, we present a phase-field approach of multicomponent and multiphase to investigate the Janus droplet formation via thermally induced phase separation. The crucial kinetics for the formation of Janus droplets consisting of two polymer species and a solvent component via an interplay of both diffusion and convection is considered in the Cahn–Hilliard–Navier–Stokes equation. The simulation results of the phase-field model show that unequal interfacial tensions between the two polymer species and the solvent result in asymmetric phase separation in the formation process of Janus droplets. This asymmetric phase separation plays a vital role in the establishment of the so-called core–shell structure that has been observed in previous experiments. By varying the droplet size, the surface tension, and the molecular interaction between the polymer species, several novel droplet morphologies are predicted in the development process of Janus droplets. Moreover, we stress that the hydrodynamics should be reckoned as a non-negligible mechanism that not only accelerates the Janus droplet evolution but also has great impacts on the coarsening and coalescence of the Janus droplets.

## 1 Introduction

The Janus droplet is an anisotropic material that is composed of two hemispheres possessing different physical, chemical, biological, and electric properties.^1−7^ Due to its anisotropic physicochemical interaction with the surrounding fluid, the Janus particle has captured extensive scientific attention and exhibits an extraordinary potential in many fields, such as display,^8,9^ microsensing,^6,10,11^ drug delivery,^12−14^ and micromotor.^15−20^ Pertaining to the prospect for the broad application of Janus droplets, several manufacturing methods, including droplet microfluidics,^1,21^ flow lithography,^22^ electrohydrodynamic co-jetting,^23,24^ and micro-molding,^25,26^ have recently been explored. Besides these above-mentioned effective fabrication techniques, Janus droplets can also be produced via thermally induced phase separation (TIPS), which has been widely investigated and utilized due to its convenient setup, high productivity, and low equipment cost.^27−29^ However, there are several disadvantages of the TIPS method, which limit its utilization in some highly sophisticated fields, for example, high droplet polydispersity, sustainable reproducibility, and diverse morphologies.^27^ In order to improve the stability for the production of the Janus particles via TIPS, it deserves more extensive studies to understand the kinetics for the formation of the Janus particles.

In previous research studies, many complex systems are experimentally investigated for both inorganic^30−33^ and organic^12,34−39^ materials. Considering the complicated recipes adopted, as well as the huge amount of adjustable material parameters, such as surface tension, viscosity, critical temperature, and diffusivity, it is almost impossible to avoid repetitive labor works for achieving a comprehensive understanding of the final morphologies of the Janus droplets. Meanwhile, the crucial but transient intermediate states during the Janus droplet evolution are highly associated with the kinetics of the system, which can be strictly restricted by the experimental methods and equipment. With the advance in computational technology, the material simulation methods provide us another prospect to carefully heed the behaviors of Janus particles. For instance, the hydrodynamics of the Janus droplet and its stability have been simulated via fluid dynamic methods, including the Lattice–Boltzmann method^40−44^ and the arbitrary Lagrangian–Eulerian method.^45^ The self-assembly properties of the Janus droplets are mainly discussed with the help of molecular dynamics,^2,46−52^ dissipative particle dynamics,^53−57^ and Brownian dynamics.^58−62^

In this work, we adopt the phase-field method to elucidate the Janus formation via TIPS, which has been widely introduced to simulate the phase separation of several multi-component systems.^63−67^ Most crucially, the microscopic material evolution can be investigated in detail by solving a series of rigorous governing equations obeying thermodynamic principles. Meanwhile, the microscopic mass transfer during the phase separation inside the Janus droplet, such as diffusion and convection, can be extensively analyzed, which may be difficult to capture under microscopes. Moreover, by adjusting key parameters, including the droplet size, initial polymer composition, interfacial tension, and molecular interaction of the Janus system, we can scrutinize the underlying mechanisms that dominate the kinetics of the Janus droplet formation. Taking the advantages of computational simulation, several interesting morphologies are observed, which may help to design and manipulate the micro-droplet with complex structures for applications in biomedical, pharmaceutical, and food industries. In addition, we explore the crucial impact of hydrodynamics on the coarsening and coalescence mechanisms of the Janus droplets, which will shed light on the control of droplet polydispersity and help to design optimum conditions for the production of Janus particles.

## 2 Numerical Model

In this work, we apply a phase-field model of Cahn–Hilliard-type coupling with the Navier–Stokes equations to simulate the formation process of Janus droplets. Janus droplets have been experimentally observed in polymer solutions consisting of polymer species A and B, and a solvent species (like water) with molecular weight much less than those of A and B.^33^ A Janus particle usually has two distinct faces that are immiscible with each other as well as with the solvent-rich matrix. Each face of the Janus particle is composed of distinct polymer species.^28^ The space- and time-dependent composition of polymer A, polymer B, and the solvent is defined as *c*_1_(**x**, *t*), *c*_2_(**x**, *t*), and *c*_3_(**x**, *t*), respectively, and is subjected to the constraint of the incompressible condition ∑_*i*=1_^3^*c*_*i*_ = 1.

The starting point of our model is the free energy functional of the Cahn–Hilliard type,^68^ which reads
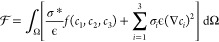
1where Ω indicates the spatial domain occupied by the system. The first term inside the bracket denotes the bulk free energy density, with σ* being the characteristic surface tension. The modeling parameter ϵ controls the interface width between different phases. The second term depicts the gradient energy density, where the coefficient σ_*i*_ is related to the interfacial tension between the immiscible phases in the ternary A–B–solvent phase diagram.

To obtain the phase diagram for the ternary system, we adopt the Flory–Huggins model^69^ with the following free energy density formulation
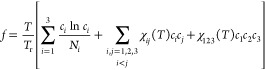
2Here, *T* denotes the temperature, *T*_r_ is a reference temperature that is chosen to be the half of the critical temperature *T*_c_ for the binary A–B miscibility gap, and *N*_*i*_ indicates the degree of polymerization for component *i*. The Flory parameters χ_*ij*_ and χ_*ijk*_ are supposed to be temperature dependent as follows^70,71^
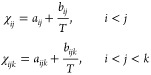
3The degree of polymerization *N*_*i*_ and the temperature coefficients for the Flory parameters *a*_*ij*_, *a*_*ijk*_, *b*_*ij*_, and *b*_*ijk*_ are chosen to be consistent with the following experimental observations: (i) when the temperature is greater than the critical temperature *T*_c_, that is, *T* > *T*_c_, polymers A and B are well miscible with each other and form a homogeneous droplet. The homogeneous droplet consisting of polymers A and B is immiscible with the solvent matrix, which is attributed to a relatively strong repulsive interaction between the solvent and the two polymer species. (ii) As *T* < *T*_c_, the repulsive force between polymers A and B increases with a reduction in temperature. The enhanced repulsive force at low temperatures leads to a miscibility gap involving polymer A-rich and polymer B-rich phases, which results in the phase separation inside the homogeneous droplets formed above the critical temperature. Such kind of features [(i) and (ii)] for the phase diagram can be found in the ternary system of hexane–perfluorohexane–water^33^ and many other ternary polymer solutions. The parameters *N*_*i*_, *a*_*ij*_, *b*_*ij*_, *a*_*ijk*_, and *b*_*ijk*_ for modeling the ternary phase diagram are tabulated in Table 1.

**Table 1:** Simulation Parameters for the Bulk Free Energy Density

parameters	description	unit	value
**N** = (*N*_1_, *N*_2_, *N*_3_)	degree of polymerization		(5, 5, 1)
**a** = (*a*_12_, *a*_13_, *a*_23_, *a*_123_)	temperature coefficient of the Flory parameters		(−1.33, 0.0, 0.0, 5.0)
**b** = (*b*_12_, *b*_13_, *b*_23_, *b*_123_)	second coefficient of the Flory parameters	K	(3.33, 4.2, 4.2, −8.0)

The phase diagram is constructed by solving the equation system for the binodal composition pair **c**^eq^ = (*c*_1_^eq^,*c*_2_^eq^,*c*_3_^eq^) and **c**^eq*^ = (*c*_1_^eq*^,*c*_2_^eq*^,*c*_3_^eq*^) as
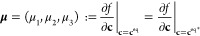
4
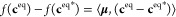
5

**Figure 1:**
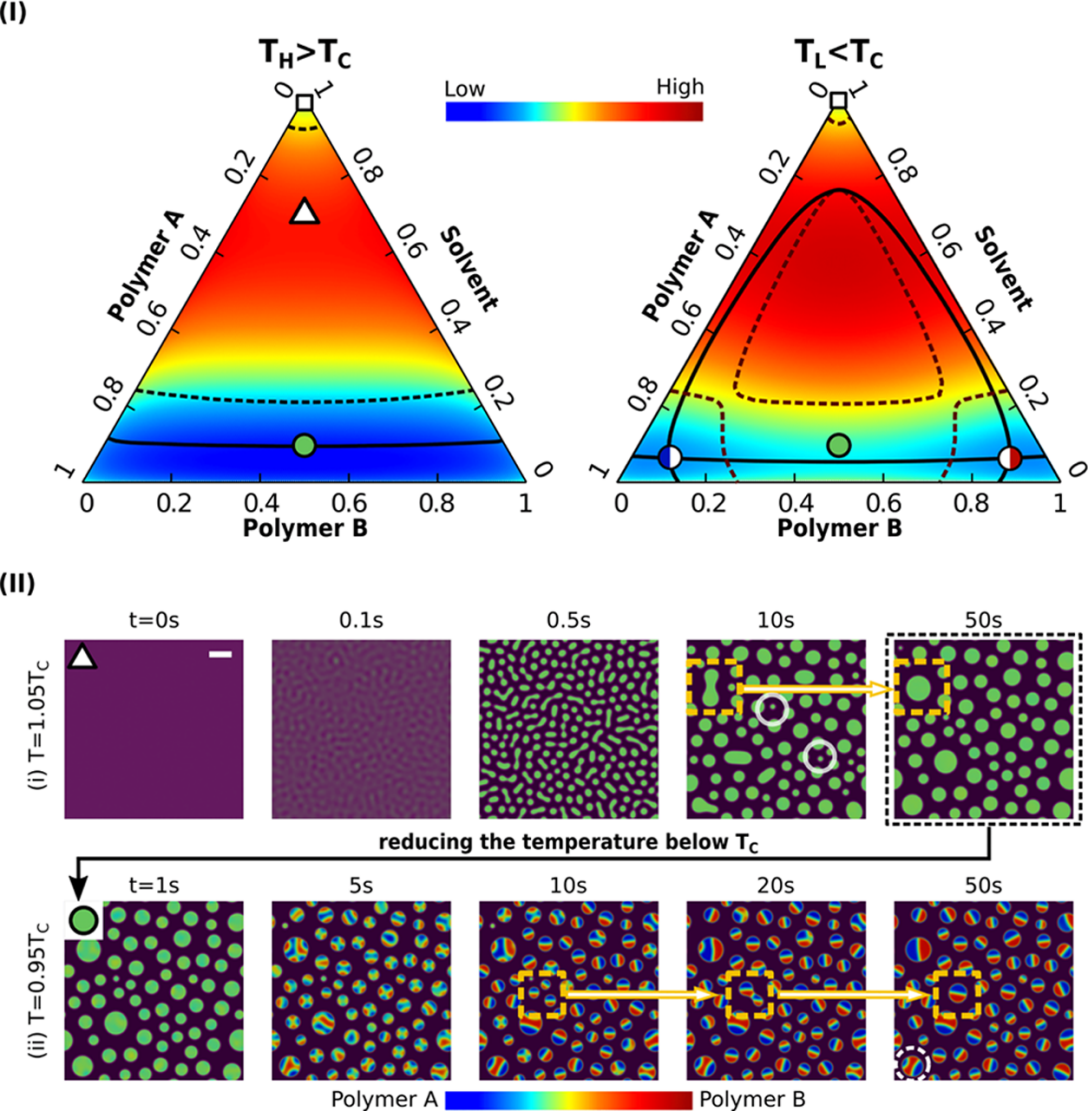
(I) Left: Ternary phase diagram of the polymer A–polymer B–solvent system at *T* = *T*_H_ (*T*_H_ = 1.05*T*_c_). The magnitude of the free energy density is described by the heat map (red: high; blue: low). The binodal and spinodal compositions are depicted by the dark red solid lines and dashed lines, respectively. The white triangle indicates the initial compositions with *c*_1_ = *c*_2_ = 0.15. The equilibrium compositions of the droplet and matrix are labeled with the green circle and the white square, respectively. Right: Phase diagram at *T* = *T*_L_ (*T*_L_ = 0.95*T*_c_). The green circle corresponds to the initial homogeneous composition in the droplet resulting from the phase separation at *T* = *T*_H_. The Janus symbols mark the equilibrium compositions in each Janus hemispheres. (II) Upper row: Microstructure evolution for the production of homogeneous polymer droplets at *T* = *T*_H_. Lower row: Formation of Janus particles via spinodal decomposition when reducing the temperature to *T* = *T*_L_. The simulation domain size is 400 μm × 400 μm. In this work, all the white scale bars denote 50 μm and each color bar scales the polymer composition, if not specified.

The spinodal composition **c**^*s*^ = (*c*_1*s*_, *c*_2*s*_, *c*_3*s*_) is defined by the locus of
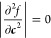
6Two typical phase diagrams at temperatures greater and less than *T*_c_ are shown in Figure 1I. The binodal and spinodal lines are depicted by the solid and dashed lines, respectively, in the contour plot for the bulk free energy density.

The temporal evolution for the compositions follows the equations
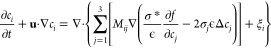
7
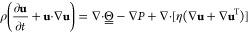
8
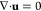
9Here, the mobility *M*_*ij*_ is assigned with the Onsager’s relationship^72,73^ as *M*_*ij*_ = *M*_0_*c*_*i*_(δ_*ij*_ – *c*_*j*_) (see the derivation in ref (74)), in which δ_*ij*_ is the Kronecker delta. The parameter *M*_0_ scales the mobility and is formulated as ∑_*i*=1_^3^*D*_*i*_*c*_*i*_, where *D*_*i*_ stands for the diffusivity of pure component *i*. In this work, we assume *D*_1_ = *D*_2_ = *D*_3_ = 1.0 × 10^–9^ m^2^/s. Moreover, the Gaussian white noise term ξ_*i*_ for the diffusion flux is essential to trigger the spinodal decomposition, which follows ⟨**ξ**_*i*_(***x***,*t*),**ξ**_*i*_(***x***′,*t*′)⟩ = *A*_ξ_^2^δ(***x*** – ***x***′)δ(*t* – *t*′), where *A*_ξ_ is the amplitude of the perturbation. Concerning the hydrodynamic effect in the system, we use incompressible Navier–Stokes equations (eqs 8 and 9) to calculate the flow velocity **u** in the ternary system. The parameters ρ, η, and *P* denote the fluid density, viscosity, and pressure, respectively. The surface tension effect is coupled into eq 8 by the Korteweg stress tensor as
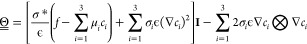
10Here, μ_*i*_ stands for the curvature-related chemical potential of component *i* and is calculated as ∂*f*/∂*c*_*i*_ – 2σ_*i*_ϵΔ*c*_*i*_. Moreover, the surface tension force in the Navier–Stokes equation is simplified as
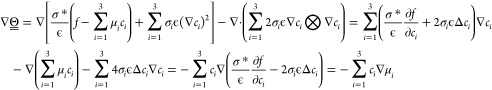
11

### 2.1 Non-dimensionalization

All the physical parameters are non-dimensionalized by the characteristic length x* = 2 × 10^–10^ m, reference surface tension σ* = 1.0 × 10^–2^ N/m, and diffusivity D* = 1 × 10^–9^ m^2^/s. Thereafter, we have the following scaling factors for the physical parameters, as shown in Table 2 (more details on non-dimensionalization are available in ref (75)).

**Table 2:** Scaling Factors for Physical Parameters

parameters	description	calculation	scaling factor
x*	characteristic length		2 × 10^–10^ m
σ*	characteristic interfacial tension		1 × 10^–2^ N/m
D*	characteristic diffusivity		1 × 10^–9^ m^2^/s
t*	time	x*^2^/D*	4 × 10^–11^ s
η*	dynamic viscosity	σ* x*/D*	2 × 10^–3^ Pa ·s
u*	velocity	D*/x*	5 × 10^0^ m/s
f*	free energy density	σ*/x*	5 × 10^7^ J/m^3^
ρ*	density	σ* x*/D*^2^	2 × 10^6^ kg/m^3^
P*	pressure	σ*/x*	5 × 10^7^ Pa

Substituting the scaling factors into the CHNS model (eqs 7–9), we obtain
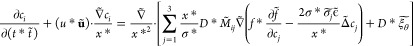

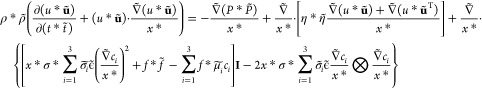

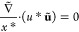
12

After simplification, the non-dimensionalized form of the Cahn–Hilliard–Navier–Stokes equation reads
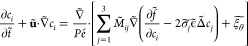

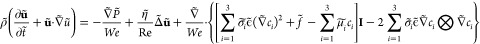

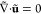
13The dimensionless quantities *Re*, *We*, and *Pé* are calculated as
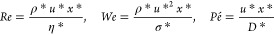
14As we are interested in the phase separation coupled with fluid flow, the convection and diffusion processes become comparable. In other words, the relaxation time for the thermodynamic process (x*)^2^/D* is in the same magnitude with hydrodynamics x*/u*, which gives rise to the Péclet number *Pé* = u*x*/D* = 1.0, while *Re* and *We* vary with the magnitude of the hydrodynamic effect.

In our work, the finite difference method and the explicit Euler scheme are implemented to solve the evolution equations eq 13 with the equidistant Cartesian mesh. Periodic boundary conditions are applied for the composition and fluid velocity.

## 3 Results and Discussion

### 3.1 Formation of Janus Droplets

In the following discussions, we consider the scenario where the phase separation via the diffusion process dominates the Janus droplet formation and the weak convection can be neglected as reported.^76^ Initially, at the temperature *T* = 1.05*T*_c_, a homogeneous solution of polymers A and B is dissolved in the abundant solvent (*c*_1_/*c*_2_/*c*_3_ = 15/15/70). The initial composition of the ternary polymer solution is marked by the triangle symbol in Figure 1I. As shown in the phase diagram, this initial composition is located between the black dashed lines, that is, inside the spinodal region. A Gaussian white noise with amplitude *A*_ξ_ = 0.1 for the diffusion flux of all components, as depicted by eq 13, is used to model the composition fluctuation. Triggered by the composition fluctuation, phase separation begins and polymer-rich droplets (light green) start to form and grow from the solvent-rich matrix (dark purple), as illustrated in Figure 1IIi. With time, these droplets coarsen with each other dominated by the Ostwald ripening effect due to their radius difference. Meanwhile, the coalescence of relatively large droplets is observed as highlighted by the dashed square. Consequently, dispersed droplets with various sizes are obtained. The equilibrium composition in the droplets is marked by the green circle on the binodal line in the left phase diagram. Because at *T* > *T*_c_, polymers A and B are miscible with each other, the Janus particle is not observed at this temperature. As demonstrated in the following section, the droplet with miscible polymers A and B formed at *T* > *T*_c_ is the precursor for the formation of the Janus droplet.

Next, we reduce the temperature till *T* = 0.95*T*_c_, where the miscibility gap between the two polymer species begins to appear, which results in the spinodal decomposition inside the precursor polymer droplet. To simulate the Janus droplet formation and to ensure a stable three-phase region, we assign the following interfacial tension parameters σ_1_ = σ_2_ = 0.64 and σ_3_ = 3.6. As presented in the snapshots of Figure 1IIii, at *t* = 5 s, the two polymer species get separated to form a polymer A-rich region (blue) and a polymer B-rich region (red). These two regions are divided by the light green interfaces. Accompanied by the proceeding spinodal decomposition, the interfaces between the blue and the red regions are stabilized (see the snapshot at *t* = 10 s). Decided by the wetting effect at the triple junction involving the polymer A-rich phase, polymer B-rich phase, and the solvent-rich phase, Janus droplets with *r* 15 μm are generated at *t* = 10 s. Afterward, the phase separation subsides, and the minimization of free energy functional is mainly manifested by the reduction in the interfacial energy. Then, the Ostwald ripening effect and coalescence come on to the stage. For instance, the tiny droplet in the white circle vanishes, because of its huge curvature difference from its surrounding large droplets. As highlighted by the orange-dashed squares in Figure 1IIii, three tiny Janus droplets coalesce into a large one due to their relatively narrow apart distances. Moreover, a large Janus droplet with a transient hamburger structure^77,78^ is captured in the white-dashed circle at *t* = 50 s. This observation indicates that the size of the precursor polymer droplet plays an important role in the morphological evolution of the Janus droplet, which will be discussed in the following sections.

### 3.2 Radius and Initial Composition

**Figure 2:**
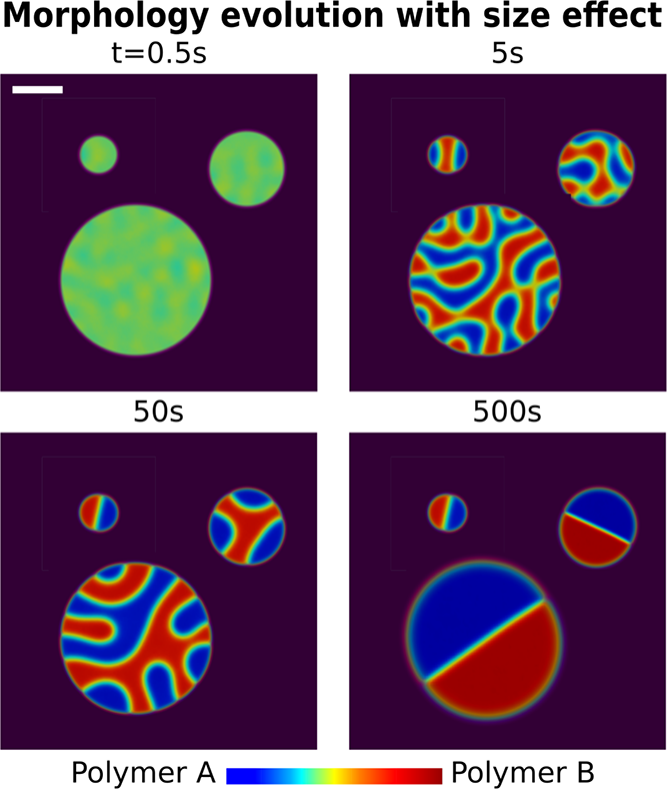
Janus droplet formation with 3 initial radii: 20, 40, and 80 μm. In simulations, all droplets are placed in their individual domains and are stitched in a domain for a better comparison.

In the manufacturing process of Janus particles, the final product with different sizes can be achieved by controlling the holding time at *T* = 1.05*T*_c_ above the critical temperature. As shown in Section 3.1, the Ostwald ripening effect together with the droplet coalescence results in precursor droplets with various sizes. However, with the descending temperature, the phase separation of polymers A and B cannot simultaneously engender the perfect Janus droplet for differently sized precursor droplets. In order to have a heedful look at this size effect, we place three precursor droplets with the initial radius *r*_0_ = 20, 40, and 80 μm in the solvent-rich matrix and reduce the temperature to *T* = 0.95*T*_c_. As shown in Figure 2, the smallest droplet with *r*_0_ = 20 μm forms a perfect Janus at 50 s, while the largest precursor droplet with *r*_0_ = 80 μm spends 20 times longer for the transformation into a perfect Janus particle. The reason for this size effect can be explained by viewing the intermediate stage of the Janus droplet development.

As depicted in Figure 2, more complex bicontinuous structures are produced inside the relatively large droplets by phase separation. The final perfect Janus droplet is formed via the coalescence of the tortuous bicontinuous structure. As demonstrated in the third snapshot of Figure 2, for all the three phases, namely, the blue-highlighted polymer A-rich phase, the red-colored polymer B dense phase, and the matrix, the equilibrium compositions are reached. Hence, at this stage, the phase separation plays hardly any role now and the Ostwald ripening is the main mechanism for the Janus droplet evolution. According to the classic theory of the Ostwald ripening,^79−81^ the mean average radius of the phase separation structure follows the quintessential LSW (Lifshitz–Slyozov–Wagner) power law r*®* ∼ *t*^1/3^. Therefore, the transformation of the droplet with *r*_0_ = 80 μm into a Janus shape takes about 4^3^ times longer than the tiny one with *r*_0_ = 20 μm.

**Figure 3:**
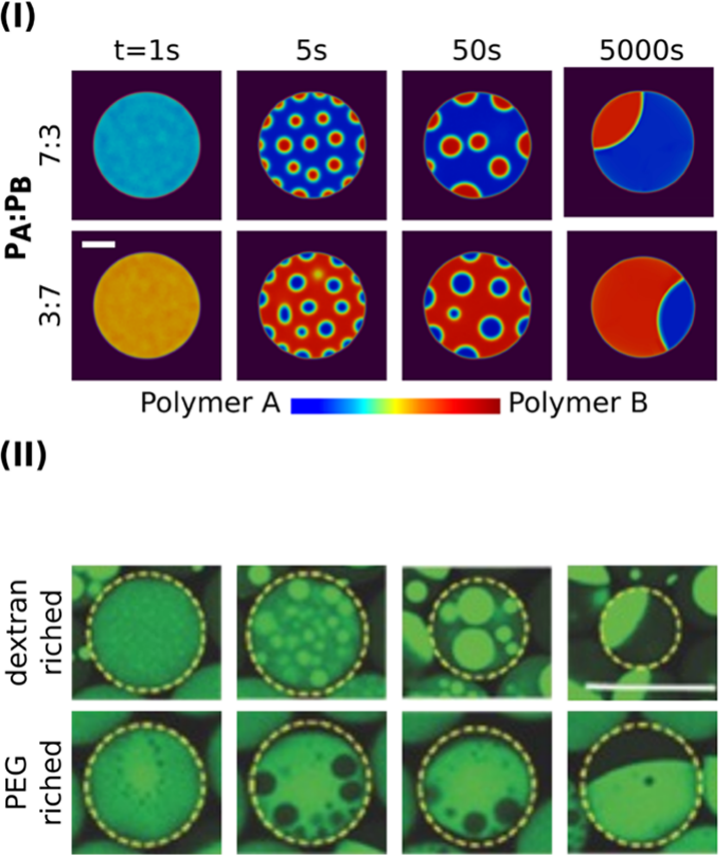
(I) Morphological evolution via sub-droplet coalescence by unequal initial polymer A and B composition ratios; upper panel: polymer A/B = 3:7; lower panel: polymer A/B = 7:3. The initial droplet size is 80 μm. (II) Confocal microscopy images of Janus particle formation by the sub-droplet coalescence in the dextran–PEG (green dyed)–water system. Reproduced with permission from ref (82) copyright ⓒ 2016 WILEY-VCH Verlag GmbH & Co. KGaA, Weinheim.

In some experiments,^82−85^ non-equal compositions of polymers A and B are adopted to synthesize different droplet morphologies, for example, vesicle, uneven Janus, and so forth. In Figure 3I, we perform simulations with unequal polymer compositions, namely, *c*_1_/*c*_2_ = 3:7 and *c*_1_/*c*_2_ = 7:3, for a precursor droplet with *r*_0_ = 80 μm. Because the composition ratio between the two polymers is off 5:5, in lieu of bicontinuous structures, sub-droplets are engendered inside the precursor droplet via the spinodal decomposition. To minimize the interfacial energy, those sub-droplets merge into a joint phase. This routine for the Janus droplet formation is in good agreement with the confocal microscopy images for the dextran–PEG–water system, as illustrated in Figure 3II. It is also noteworthy that the interface between polymers A and B in our simulation (7:3 in Figure 3I) has a different convexity from the experiment (PEG-riched case in Figure 3II). As observed in the hexane−perfluorohexane Janus system,^33^, the Janus structure is extremely sensitive to the composition-dependent interfacial tension parameter σ_*i*_. Thus, the slight polymer composition changes can result in the huge difference in the interface convexity.

### 3.3 Interfacial Tension

**Figure 4:**
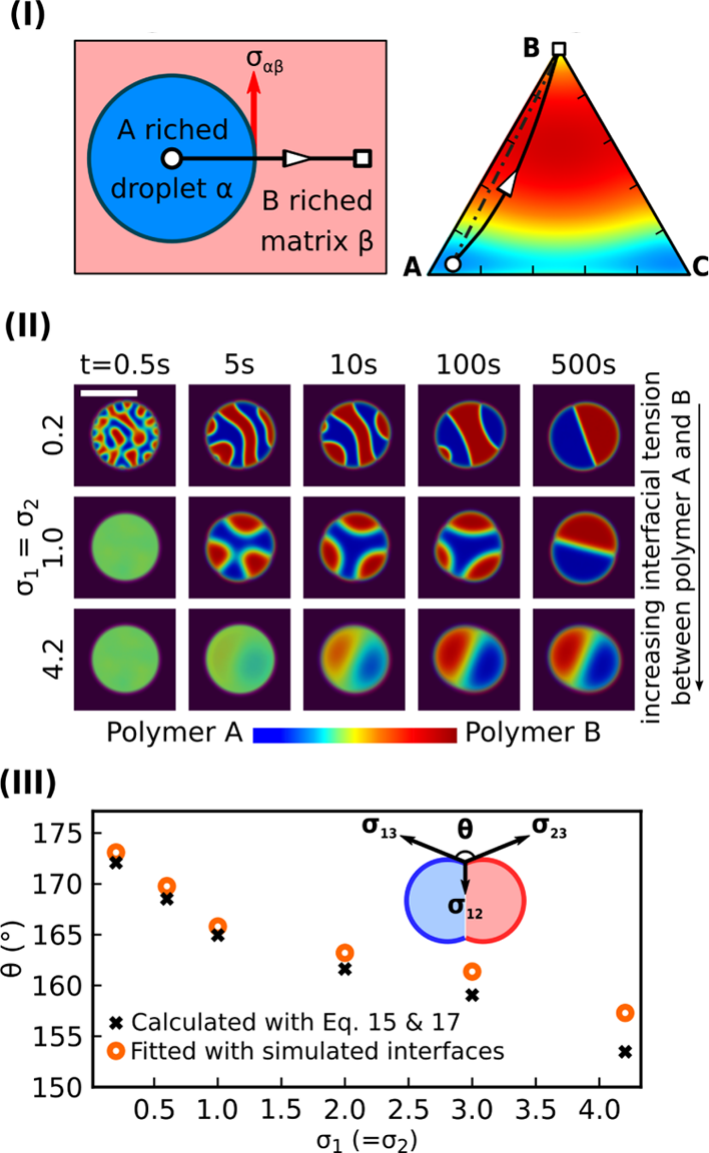
(I) Left: Equilibrium interfacial tension σ_αβ_ of the A-rich droplet α and B-rich matrix β with the sharp interface model. Right: schematic integral routine with the black open arrow on the phase diagram for the calculation of σ_αβ_ in the Cahn–Hilliard model. (II) Formation of the Janus particle via the spinodal decomposition with the increase in interfacial tension parameters σ_1_ = σ_2_. The initial droplet sizes are 20 μm. (III) Contact angle θ between two hemispheres versus σ_1_ = σ_2_. Black cross: calculated with eq 17; orange open dot: fitted with simulation.

In the previous part, we elucidated the formation of Janus droplets via liquid–liquid phase separation, which transforms the bulk free energy of the mixture into the interfacial energy. The latter one determines the pattern of the microstructure around the triple junction. In this section, we address the microstructure pattern at the region of the triple junction by varying the interfacial tension. In experiments, the adjustment of interfacial tensions is achieved by adding various surfactants into the system.^33^ As a rule of thumb, the surfactant quantity less than 0.1% v/v has a minor effect on altering the ternary phase diagram.^76^ In our simulations, the effect of specific surfactants can be mimicked by changing the interfacial tension parameter σ_*i*_. To be consistent with the sharp interface model, the interfacial tension between the component A-rich phase and the component B-rich phase in the Cahn–Hilliard approach (see the right column of Figure 4I) is calculated as
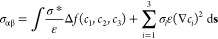
15where **s** delineates the integral routine that follows the energy minimum principle. As displayed by the solid arc with a white open arrow in Figure 4I, the integration starts from the white open dot with one bulk equilibrium composition (*c*_1_^eq^,*c*_2_^eq^,*c*_3_^eq^) inside phase α to the other bulk equilibrium composition (*c*_1_^eq*^,*c*_2_^eq*^,*c*_3_^eq*^) inside phase β. The term Δ*f* is defined as
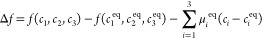
16which measures the excess free energy density referring to the equilibrium state *f*(*c*_1_^eq^,*c*_2_^eq^,*c*_3_^eq^) + ∑_*i*=1_^3^μ_*i*_^eq^*c*_*i*_^eq^. From this expression, the interfacial tension of the system not only is decided by the interfacial tension parameters σ_*i*_ but also relies on the bulk free energy density of the mixture. Owing to the addition of the third component (C in this example), the integral routine at the equilibrium state does not take the dot-dashed straight line from α to β in the phase diagram but follows the deterministic path that corresponds to the fundamental energy minimization principle as addressed by the Cahn–Hilliard equation (eq 7). In this way, the interfacial tensions in the ternary system can deviate largely from the one in the binary system, crucially depending on the Flory parameters χ_*ij*_ and χ_123_. It appears that the addition of the third component dramatically increases the complexity for measuring and calculating the interfacial tension. In most cases, there is a paucity of experimental data for the interfacial tensions to validate the model. Here, we systematically discuss the effect of the interfacial tension on the pattern formation of the Janus particles.

First, we analyze the interfacial tension parameter σ_*i*_ affecting the formation of the Janus droplet. The interface tension parameters σ_1_ and σ_2_ both vary from 0.2 to 4.2, while all other parameters are the same as that in Section 3.2. This setup can be comprehended as introducing a tiny amount of a surfactant that can drastically reduce the interfacial tension of the system, while the phase diagram is not altered. As displayed in Figure 4II, an increase in σ_1_ and σ_2_ results in different morphological evolutions during the phase separation. For instance, the threefold symmetric droplet is observed in the setup with σ_1_ = σ_2_ = 1.0, which shows high similarities with the experimental observations for the patchy droplet structures of self-assembly materials.^86^ With an increase in σ_1_ and σ_2_, the time expense for the formation of the Janus droplet via the Ostwald ripening is sharply reduced and the final contour of the Janus droplet transforms from a joint sphere to a quasi-ellipse. In Figure 4III, the contact angle θ between two Janus hemispheres is measured with two different methods. (1) Orange open dots: the interfaces between the Janus hemispheres and the solvent matrix are extracted from the simulation by the criterion: *c*_1_ = 0.5 for the polymer A hemisphere and *c*_2_ = 0.5 for polymer B. As sketched by the inset in Figure 4III, we fit the interfaces between the polymer A-rich hemisphere and the solvent matrix and between the polymer B-rich hemisphere and the solvent matrix with circles. The interfaces intersect at the triple junction where the contact angle θ is computed by the included angle of the two tangent lines of the fitted circles. (2) Black crosses: with eq 15, the three interfacial tensions form the so-called Neumann triangle relation, which constrains the θ at equilibrium as
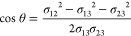
17Showing good agreement in these two methods, the contact angle θ decreases by enlarging the interfacial tension parameter σ_1_ (=σ_2_). The comparison not only indicates the droplet contour changing from Janus (θ ∼ 180°) to quasi-dumbbell structure (θ ∼ 155°) controlled by the interfacial tension but also validates the Cahn–Hilliard model for the Janus droplet system.

**Figure 5:**
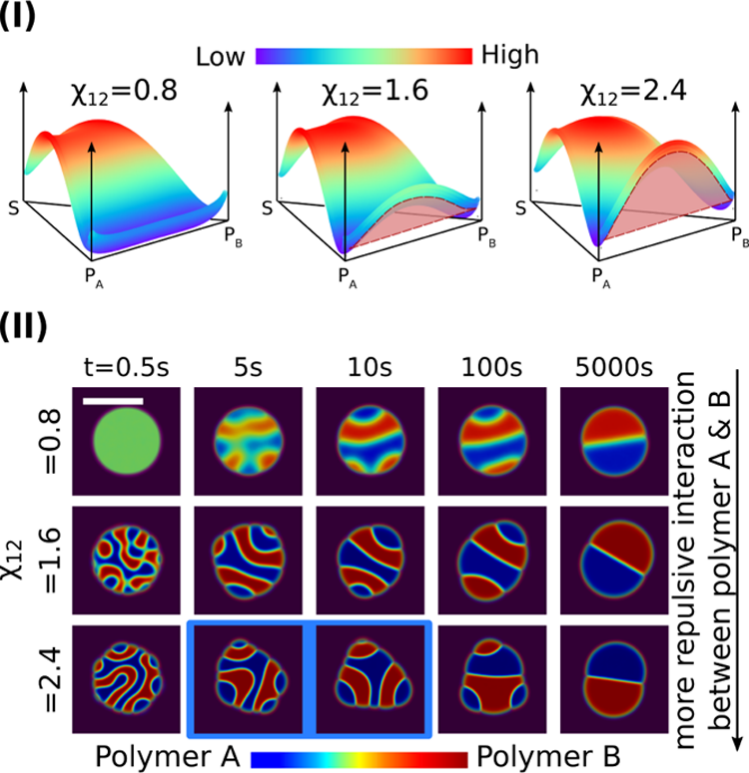
(I) Free energy density at *T* = 0.95*T*_c_ for different Flory parameters χ_12_ between polymer A and polymer B. The light red shadow regions mark the miscibility gaps. (II) Formation of the Janus droplet by enhancing χ_12_. The blue highlighted snapshots display the transient pentagon and triangle droplet shapes. The initial droplet size is 20 μm.

Second, we assign a different Flory parameter χ_12_ with the same interfacial tension parameters σ_1_ = σ_2_ = 1.0 in the simulation. This setup may be achieved by applying different polymer species in the system. As demonstrated in the free energy landscapes in Figure 5I, an increase in χ_12_ not only modifies the interfacial tension between polymers A and B but also broadens the miscibility gap, as depicted by the light red shadow regions. Hence, a more pronounced phase separation accelerating the production of polymer A/B interfaces is expected for a larger value of χ_12_, as demonstrated in Figure 5II. Most interestingly, in the blue highlighted simulation snapshots of Figure 5II, intermediate droplets with polygon and triangle shapes are observed. This observation implies that the thermally induced liquid–liquid phase separation may have the potential to synthesize droplets with some special morphologies, which shall be investigated in the future.

### 3.4 Asymmetric Phase Separation

In the previous sections, the Flory parameters χ_13_ and χ_23_ are assumed to be the same, so do the interfacial tension parameters σ_1_ = σ_2_. These setups usually give rise to symmetrical kinetics for the formation of the Janus droplet, that is, different faces of the Janus droplet and the diffusion path of distinct polymer species following identical kinetics. Actually, in most real systems, the Flory parameters of polymer A and polymer B with the solvent are not equal, for example, χ_13_ ≠ χ_23_, denoting different attractive/repulsive potential of the polymer species with the solvent molecules. In addition, the interfacial tensions between polymer species and the solvent should be distinct from each other, which may be caused by unequal interfacial tension parameters, σ_1_ ≠ σ_2_. The Janus droplet formation is attributed to the interplay of both liquid–liquid phase separation and the interfacial energy minimization, the kinetics of which is essentially affected by both the Flory parameters and the interfacial tension parameters. Thus, the distinguishing properties of polymers A and B may lead to asymmetrical kinetics for the microstructure evolution of the Janus droplet formation as well as a dissymmetric diffusion path, which will be discussed in this section.

**Figure 6:**
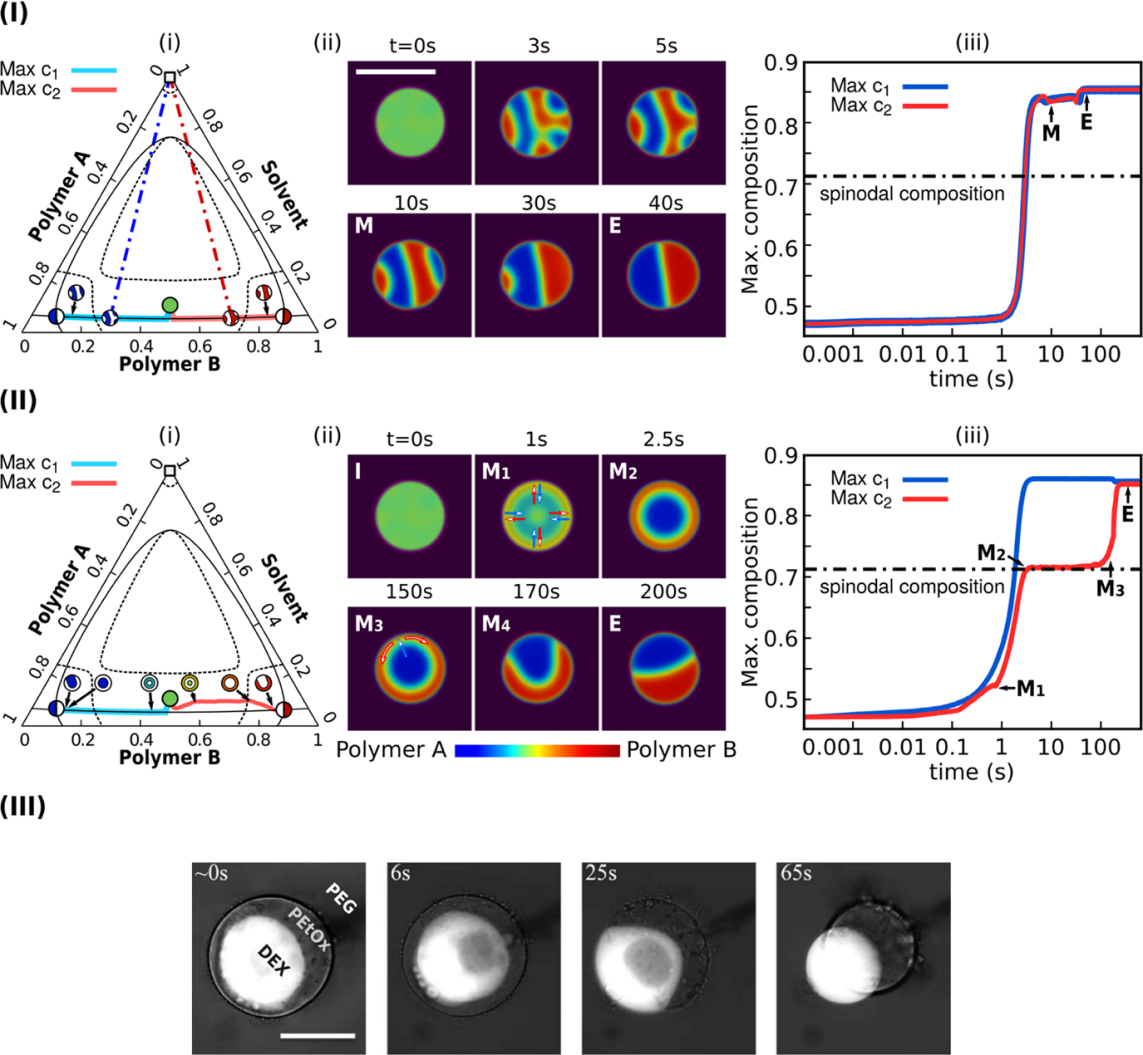
(I) Symmetric kinetics of the Janus droplet formation with the same interfacial tensions σ_13_ = σ_23_. (i) Kinetic pathway of the maximal polymer A-rich region (turquoise solid line) and the maximal polymer B-rich region (red solid line) in the phase diagram. The dot-dashed lines indicate the tie lines for the pseudo-binary equilibrium between polymer droplets and the solvent matrix; (ii) Janus droplet formation with the initial droplet size *r*_0_ = 20 μm; (iii) time sequence of the composition with maximal *c*_1_/*c*_2_ (blue/red solid line). The dot-dashed horizontal line indicates the spinodal composition. (II) Asymmetric kinetics of the Janus droplet formation with unequal interfacial tensions σ_13_ > σ_23_. The initial droplet size is 20 μm. (III) Microscopy images of Janus particle formation via the asymmetric phase separation in a three-phase system. Bright: dextran (DEX); gray: poly(2-ethyl-2-oxazoline) (PEtOx), and gray matrix: polyethylene glycol (PEG). Reproduced with permission from ref (85) copyright ⓒ 2021, American Chemical Society.

To elucidate the symmetric kinetics, we place one homogeneous droplet with *r*_0_ = 30 μm and initial composition *c*_1_ = *c*_2_ = 0.47 inside the polymer lean matrix (*c*_1_ = *c*_2_ = 0.00025). In this setup, we adopt the same interfacial tension and Flory parameters for polymers A and B, that is, σ_1_ = σ_2_ = 0.6 and χ_13_ = χ_23_ = 4.2. From eq 15, we have σ_13_ = σ_23_. Triggered by the composition fluctuation, the phase separation generates a polymer A-rich region (blue colored) and a polymer B-rich region (red colored), which comprise the bicontinuous structure resulting from the spinodal decomposition, as displayed in Figure 6Iii. The kinetics of the Janus droplet formation is characterized by tracing the maximal polymer A composition *c*_1_ (turquoise line) and the maximal polymer B composition *c*_2_ (red line) starting from the green circle in the phase diagram. As can be noticed in Figure 6Ii, once the polymer A-rich blue region forms, the turquoise trajectory of the maximal *c*_1_ falls exactly on the black solid binodal line. This overlap implies that there exist a series of transient pseudo-binary equilibrium states between the polymer A-dense blue region and the polymer lean solvent matrix (black open square). One typical exemplary pseudo-binary equilibrium is shown by the blue dot-dashed tie line. This kind of pseudo-binary equilibrium is unstable over time because of the presence of the adjacent polymer B-rich red region, which is not in equilibrium with the polymer A-dense region. Driven by the free energy minimization, the immiscible polymer A in the adjoining red region is rejected. For the low solubility of polymers in the solvent matrix, phase separation-induced mass transformation via the surrounding matrix is in vain. The precipitated polymer A from the red region can diffuse only across the A–B interface, resulting in an enrichment of polymer A in the blue-colored regions. Consequently, the maximal composition *c*_1_ continuously moves leftward along the binodal line and finally reaches the ternary equilibrium composition labeled by the blue Janus symbol in the phase diagram in Figure 6Ii. At this right stage, a perfect Janus droplet is completely formed. The same kinetics happens inside the polymer B region, because of the exactly equal interfacial tension and Flory parameters of polymers B and A. As depicted in Figure 6Iiii, we observe the same tendencies of the maximal polymer A composition (blue solid line) and maximal polymer B composition (red solid line) changing with time. The small ups and downs are in line with the coarsening and coalescence of the phases that change the curvature and consequently the composition. In this way, we denominate this kind of kinetics as “symmetric”, which denotes the identical kinetic pathways for polymers A and B.

In our second simulation, we set the interfacial tension parameters to be asymmetric for polymers A and B as σ_1_ = 1.0 and σ_2_ = 0.6. The Flory parameters are χ_13_ = χ_23_ = 4.2, as the one in Figure 6Ii. With eq 15, it can be shown that the interfacial tension between the polymer B-rich phase and solvent-rich phase σ_23_ is smaller than that between the polymer A-rich phase and solvent-rich phase σ_13_. Hence, at the very beginning of the phase separation, in order to reduce the interfacial energy, polymer B prefers to contact with the solvent matrix and encircles polymer A to prevent the formation of the interface between polymer A and the solvent matrix. Such a kinetic process leads to centrosymmetric diffusion fluxes of polymers A and B. Accordingly, an intermediate core–shell morphology is established, which has been observed in many micro-capsule systems.^37,87−90^ As shown in Figure 6IIi for the kinetic pathway in the phase diagram, the core–shell morphology is accompanied by a deviation in the red trajectory (the maximal *c*_2_) from the black solid binodal line. This deviation is explained as follows. From the intermediate state *M*_1_ to *M*_2_, due to spinodal decomposition, the blue polymer A-rich core forms and quickly reaches the equilibrium composition on the binodal line. However, in the shell region, the kinetics is totally different. Because both the polymer A-rich phase and the solvent matrix contact with the shell, there exist two interfaces, namely, the polymer A–B interface and the polymer B–solvent interface. The composition in the shell region evolves via not only the phase separation with respect to the polymer-A rich core but also the binodal decomposition pertaining to the solvent matrix. The non-equal diffusion fluxes at the core–shell interface via spinodal decomposition and at the shell–solvent interface via binodal decomposition give rise to the deviation in the maximal *c*_2_ trajectory from the binodal line in the polymer B-rich region.

In Figure 6II, between the time points *M*_2_ and *M*_3_, the maximal *c*_2_ hardly increases with time and the core–shell structure stagnates. The reason for this utterly slow morphological transformation can be explained by the kinetic pathway in the phase diagram in Figure 6IIi. At *M*_2_, the red trajectory passes through the black dotted spinodal line and the composition in the shell falls outside the spinodal region. Thereafter, the microstructure evolution is dominated by the binodal decomposition instead of spinodal decomposition. Because the former is much slower than the latter, the composition *c*_2_ in the shell region increases with time at a relatively slow rate, leading to a stagnation state from *M*_2_ and *M*_3_. Noteworthily, the core–shell structure is not the energy minimal state. In the shell region, the everlasting Gaussian composition fluctuation gives rise to small composition gradients and produces a circular diffusion for the polymer B component, leading to the breakup of the core–shell structure at *M*_4_. When the core–shell structure collapses, the interfacial tension rapidly reshapes the polymer B-rich layer into a hemisphere (at *E*), which is the final equilibrium state of the system. We name the observation in this setup “asymmetric kinetics” to make the distinction from the previous “symmetric” case, which has also been studied in many experiments,^83−85^ as the microscopic images shown in Figure 6III.

### 3.5 Fluid Dynamics

In the previous Sections 3.1–3.4, we considered the Janus droplet formation via the diffusion-controlled phase separation process and observed a stagnation stage for the intermediate core–shell microstructure in the formation process of the Janus droplet. In this section, we will shed light on the effect of hydrodynamics on the morphological evolution of the Janus particle as well as the core–shell microstructure. Considering the kinetics of the polymeric phase separation, the polymer diffusivity can vary from 10^–9^ to 10^–12^ m^2^/s, which is decided by the degree of polymerization, temperature, solvent property, and so on. Thus, when the polymer system becomes semidilute, or even dense, the diffusivity of polymer chains decreases drastically. When D* drops to the order of 10^–12^ m^2^/s, the hydrodynamic effect plays a non-negligible role compared with diffusion. As written in eq 11, the composition gradient ∇*c*_*i*_ in the inhomogeneous fluid system can propel the fluid flow via the capillary tensor term Θ in the Navier–Stokes (NS) eq 8. This mechanism is called the Marangoni effect. By adjusting the dimensionless numbers, (1) Weber number: *We* and (2) Reynolds number: *Re*, in the non-dimensionalized NS eq 13, we will show distinct hydrodynamic behaviors for the microstructure evolution of the Janus droplet.

**Figure 7:**
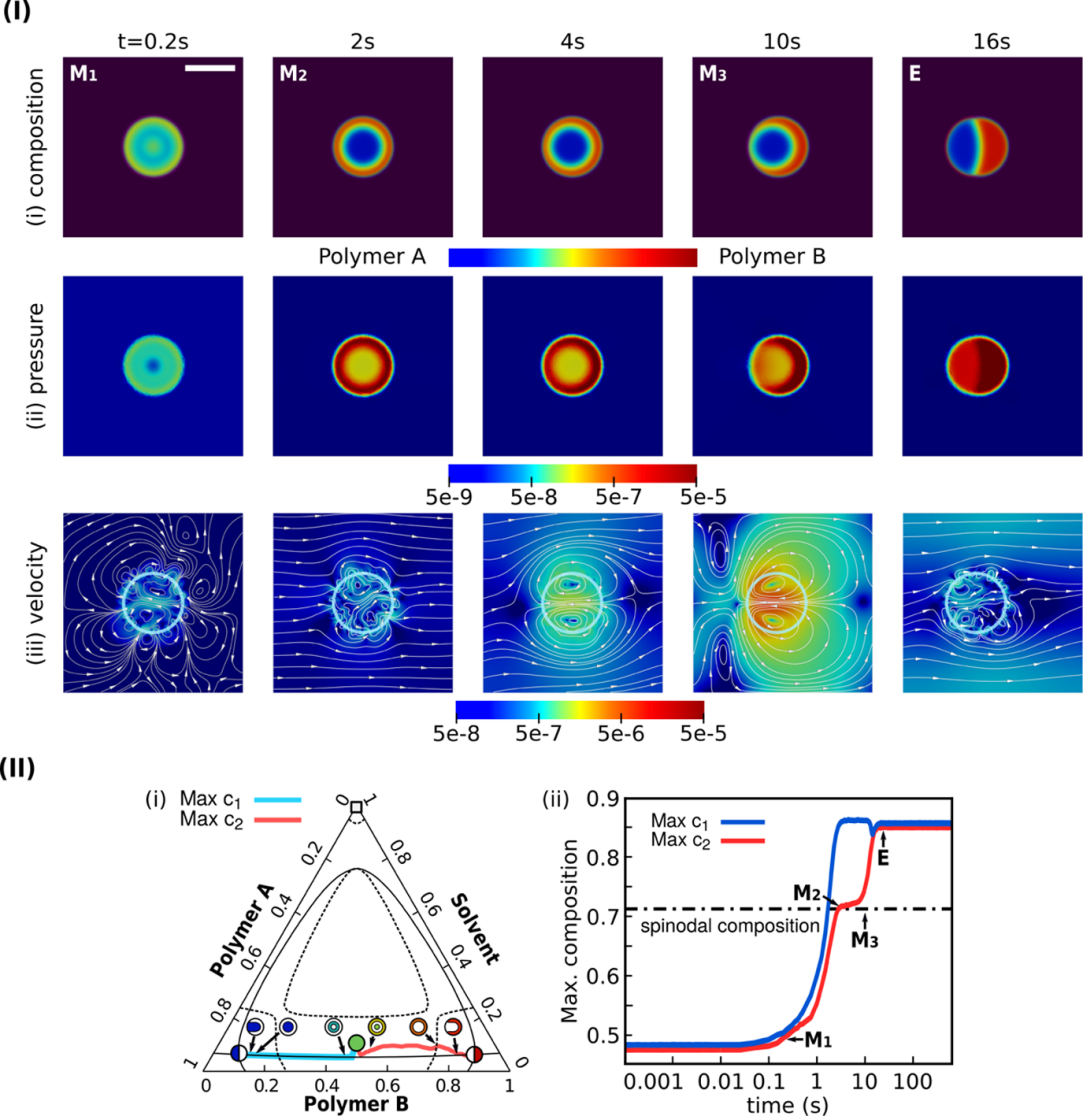
(I) Single Janus droplet formation with asymmetric interfacial tensions σ_13_ > σ_23_ and the hydrodynamic effect *We* = 1.0. The initial droplet size is 20 μm. (i) Composition field; (ii) pressure distribution; (iii) velocity field and the stream lines. The white translucent circles mark the Janus droplet interface with solvent composition *c*_3_ = 0.5. Each color bar beneath shows the respective magnitude. (II) Asymmetric kinetics of the Janus droplet with the hydrodynamic effect (*We* = 1.0) and unequal interfacial tension σ_13_ > σ_23_. (i) Kinetic pathway of the maximal polymer A/B dense region (turquoise/red solid line) in the phase diagram. (ii) Time sequence of the composition with maximal *c*_1_/*c*_2_ (blue/red solid line).

In a first setup, we perform a single droplet simulation with the asymmetric interfacial tension σ_13_ > σ_23_ as discussed in Section 3.4. To couple the hydrodynamic effect with the phase separation, the Weber number *We* and Reynolds number *Re* are both set as 1. As can be noticed in both the morphological evolution and the kinetic pathway in Figure 7, the transient core–shell structure survives 10 times shorter than the simulation without the hydrodynamics shown in Figure 6. By viewing the pressure distribution around the droplet in Figure 7Iii, the pressure has its maximum (dark red colored) in the polymer B-dense shell region, the minimum inside the solvent matrix, and the intermediate value (yellowish) in the polymer A-rich core. Here, the pressure is induced by the surface tension force subjected to the incompressible condition. In order to reduce the surface energy, the convection resulting from the pressure difference on both sides of the shell structure together with the diffusion leads to the breakup of the shell, which is faster than the morphological transformation solely via diffusion. The stagnation period of the core–shell structure around the spinodal composition lasts only several seconds in this case.

The Marangoni flow has two origins: (i) phase separation driving force. During the phase separation process, the composition has not yet reached the equilibrium value, where excess free energy density of the mixture results in the surface tension force ∇·Θ in the N–S equation. As demonstrated in eq 10, the term ∇·Θ is proportional to the chemical potential gradients, which cannot be balanced by the pressure *p* around the droplet. Consequently, the convection takes place. (ii) Minimization of the interfacial energy. The phase separation leads to the creation of new interfaces. The non-uniform curvature along the interface as well as the surface tension force enforcing the Young’s contact angle at the triple junction also gives rise to a convection. The former occurs at the early stage of the phase separation. The latter appears once new interfaces are established and dominates the evolution when the bulk composition reaches the equilibrium value. It is noteworthy that both mechanisms also exist when the Janus droplets are produced by the phase separation solely via diffusion (see Figure 1). The main difference is the way that the energy is minimized or, more suffice it to say, the energy dissipation associated with different kinetics. When the hydrodynamics is coupled, the excess free energy and surface energy can be transformed into the kinetic energy of the fluid flow. As displayed in Figure 7Iiii, the fluid velocity around the droplet increases simultaneously with the proceeding spinodal decomposition. After reaching the equilibrium composition and the breakup, the non-uniform curvature and the free energy minimization at the triple junction lead to a further increase in the fluid velocity. When the curvature becomes uniform around the Janus droplet and the contact angle at the triple junction reaches the equilibrium value, the fluid flow dissipates and tends to vanish, as shown in Figure 7I at *t* = 16 s.

**Figure 8:**
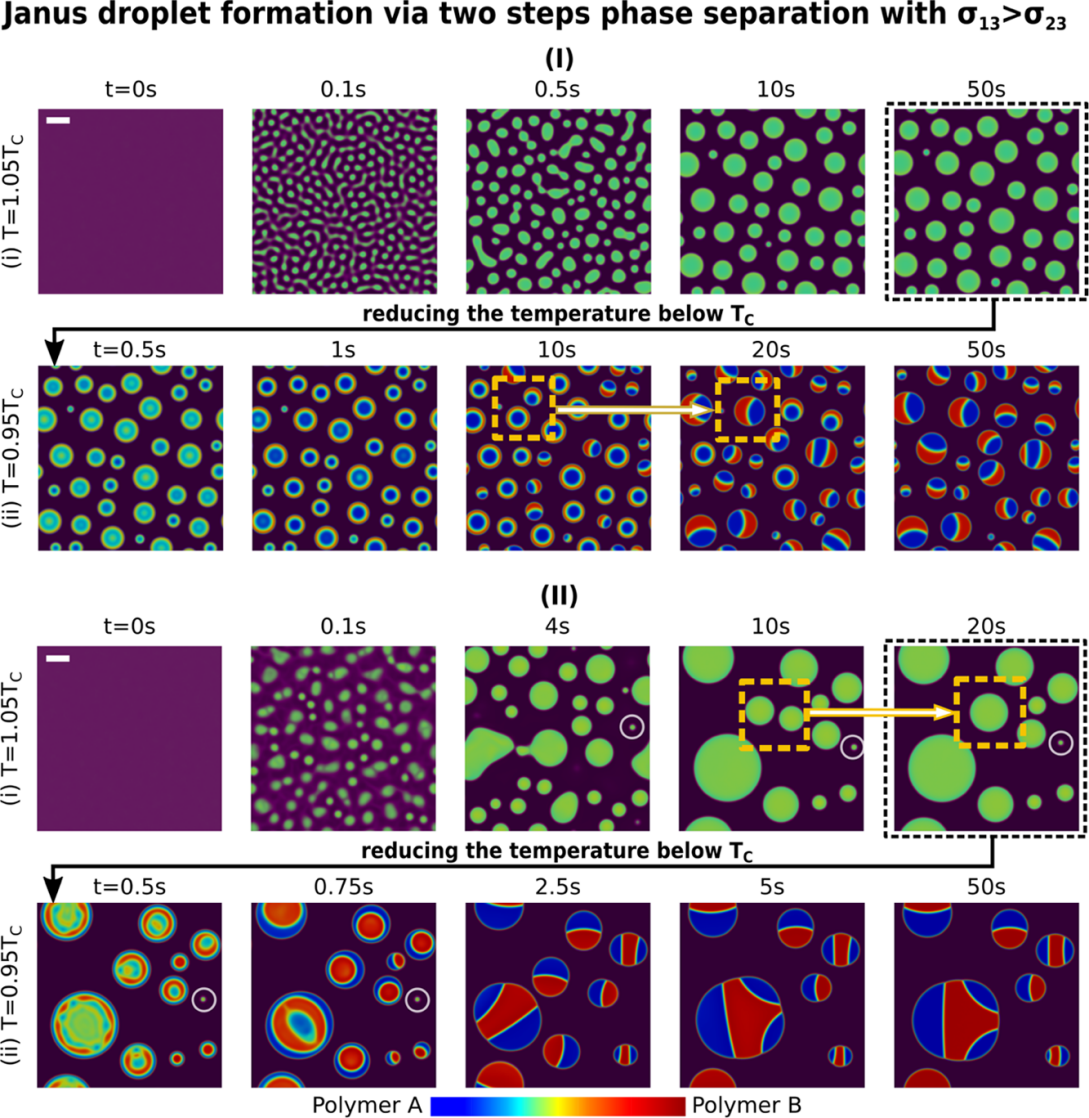
Janus droplet formation via phase separation with the surface tension of polymer A (σ_13_) larger than the surface tension of polymer B (σ_23_). (I) Spinodal decomposition coupling with a weak capillary effect with *We* = 1. Step (i): at high temperature: *T* = 1.05*T*_c_; step (ii): at low temperature: *T* = 0.95*T*_c_. (II) Spinodal decomposition coupling with a weak capillary effect with *We* = 0.01. (i) High temperature: *T* = 1.05*T*_c_; (ii) low temperature: *T* = 0.95*T*_c_. The simulation domain size is 400 μm × 400 μm.

Next, we simulate the Janus droplet formation via a two-step phase separation. At step (i), homogeneous polymer droplets are produced at *T* = 1.05*T*_c_. At step (ii), the temperature is reduced to *T* = 0.95*T*_c_, where Janus droplets are formed. Here, two magnitudes of the hydrodynamic effect are considered, namely, *We* = 1 and 0.01, to elucidate its influence on the multi-droplet system. By the definition of the Weber number, a large *We* indicates a strong capillary effect. By comparing the morphological evolution in Figure 8 with the previous case only with diffusion (Figure 1), the phase separation process is magnificently accelerated. For the phase separations at a high temperature *T* = 1.05*T*_c_, it always takes less time *t*_e_ for the droplet to reach its equilibrium concentration when the hydrodynamic effect becomes more pronounced. For *We* = ∞ (diffusion only), *t*_e_ = 8.5 s; *We* = 1.0, *t*_e_ = 2.0 s; *We* = 0.01, *t*_e_ = 0.5 s. As *T* = 0.95*T*_c_, concerning the size effect discussed in Section 3.2, droplets with similar sizes are compared. The Janus droplet production also becomes quicker with the stronger convection. For *We* = ∞, *t*_e_ = 30.0 s; *We* = 1.0, *t*_e_ = 18.0 s; *We* = 0.01, *t*_e_ = 2.25 s.

Also, the size distribution of the Janus droplet becomes more dispersed with the enhancing hydrodynamic effect. The underlying mechanism can be elucidated with the Tanaka–Golovin theory^91^ that has two important contributions to the polydispersity of the Janus droplet. On the one hand, the hydrodynamic force is produced by the overlap of the diffusion potential and propels the droplet coalescence. Hence, the droplets with relatively small apart distances are prone to impinge with each other, forming a large droplet, as illustrated in the gold dashed squares of Figure 8. On the other hand, the hydrodynamics can act as a supporter for the tiny droplet and curtails the consumption of small droplets by its large peers because of the competitive fluxes of diffusion and convection (see the discussion in refs (92) and (93)). The distinct life time of the mini-droplets circled in Figures 1 and 8 confirms the supporting effect of hydrodynamics. The pronounced coalescence effect for large droplets and the shielding effect for the small droplets result in the polydispersity of Janus droplets. This observation is also consistent with the theoretical derivation.^94^ In addition, because both the phase separation and the fluid mechanics are considered in our model, the Janus production via the microfluidic process can also be simulated and will be discussed in future works.

**Figure 9:**
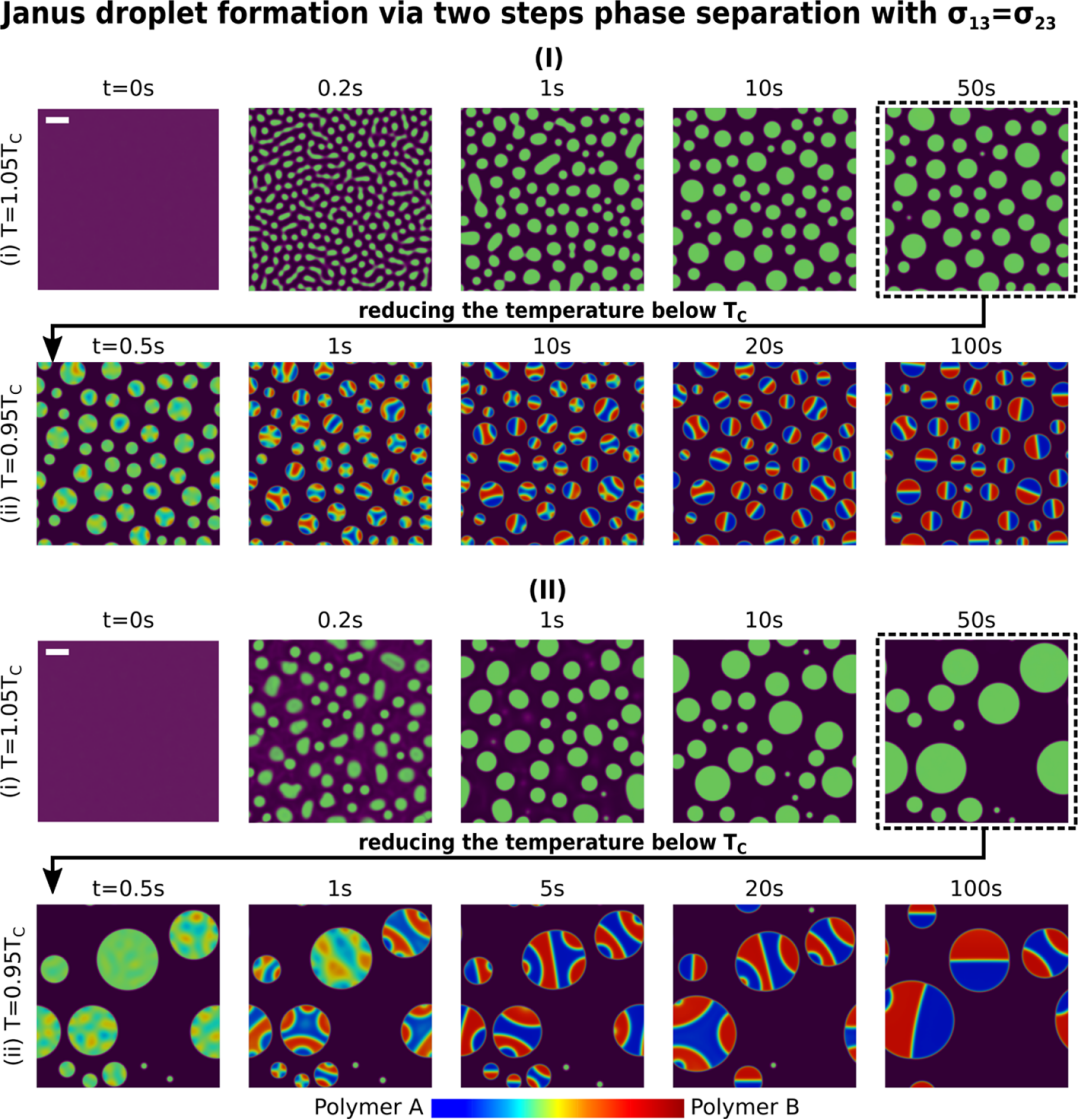
Janus droplet formation via phase separation with the surface tension of polymer A (σ_13_) equal to the surface tension of polymer B (σ_23_). (I) Spinodal decomposition coupling with a weak capillary effect with *We* = 1. Step (i): at high temperature: *T* = 1.05*T*_c_; step (ii): at low temperature: *T* = 0.95*T*_c_. (II) Spinodal decomposition coupling with a weak capillary effect with *We* = 0.01. (i) High temperature: *T* = 1.05*T*_c_; (ii) low temperature: *T* = 0.95*T*_c_. The simulation domain size is 400 μm × 400 μm.

Finally, we simulate the Janus droplet formation where the surface tensions of polymers A and B are identical. As shown in Figure 9, the perfect Janus droplets with symmetric hemispheres of A and B are produced. Similar to the previous simulations with unequal surface tensions (Figure 8), the Janus sizes increase with the increase in the hydrodynamic effect. The size distribution also turns to be more dispersed by enhancing the convection inside the system, which is attributed to the two reasons discussed in the previous paragraph: (i) the imbalanced surface tension force ∇·Θ induces convection that propels the droplet coalescence; (ii) the strong Marangoni flow also acts as a counter-force that curtails the diffusion process. Under this circumstance, the LSW mechanism plays only a subordinate role and the mean radius of the Janus droplet does not follow the 1/3 scaling law anymore.^91^

## 4 Conclusions

In summary, we have presented a multi-component and multi-phase Cahn–Hilliard–Navier–Stokes model to simulate the formation of the Janus droplet via the thermally induced liquid–liquid phase separation. By considering the initial polymer composition, Flory parameters, and the interfacial tension parameters, we elucidate the competing mechanisms for the morphological evolution, namely, (i) the diffusion-dominated phase separation process and (ii) the minimization of the interfacial energy. Due to the interplay of these two crucial factors, various transient morphologies of the droplets have been observed, including the polygon-shaped structures and patchy droplets, which need to be investigated in detail in future work. Most importantly, we stress the significance of the hydrodynamic effect. Owing to the curtailed diffusivity of entangled long polymer chains, the composition inhomogeneity-induced Marangoni effect becomes comparable with the diffusion of polymeric species. Hence, not only the phase separation process of the droplet but also the droplet coalescence is magnificently accelerated by the Marangoni flow, which results in a broad range for the size of Janus droplets. Further systematic computational studies of material properties and pattern correlations will be considered in the forthcoming researches. As the approach is already capable to be applied in three-dimensional structures, the 3D simulation of the Janus droplet will be conducted and analyzed. We believe that the present research involving Janus droplet formation via diffusion as well as fluid dynamics will shed new light on the multi-phase microfluidic manipulation technology for wide applications.
